# Treatment of Chronic Cystitis

**Published:** 1875-10

**Authors:** 


### Treatment of Chronic Cystitis.

In the course of some remarks made before the Kings Co. Medical Society, by Dr. J. W. S. Gouley, of New York, on the treatment of chronic cystitis, the doctor said that the disease is usually the consequence of some pre-existing trouble—such as may cause certain alterations of the urinary secretion—such as a foreign body in the bladder, a morbid growth, or an obstruction in the urethra. He had seen cases of chronic cystitis in middle-aged men, which could be traced to no other cause than persistent lithuria; the patients having for months, and even for years before, passed, almost daily, large quantities of crystals or agglomerations of crystals of uric arid, with great frequency of urination, vesical tenesmus, and "burning, cutting" pain along the urethral canal, the urine containing, besides, a variable quantity of pus, and sometimes of blood. This, he said, occurs most frequently among dyspeptics, and such cases of cystitis might properly be called diathetic. When this condition is discovered early, the consequent troubles can generally be averted by properly directed treatment. He recommended alteratives, laxatives, mineral acids as tonics, followed in a few days by milk alkaline waters, and then the Friedrichshalle, or the Hungarian bitter water, proper hygienic management, etc., and related illustrative cases.

In cervical cystitis with inordinate irritability he strongly advocated instillations of nitrate of silver solution, after the plan of Doctor Félix Guyon, of Paris.

The doctor spoke of the cystitis which occurs from excessive use of opium, and advised caution in the administration of this drug to patients already suffering from cystitis.

He also referred to a cervical cystitis, lasting for months, with frequent discharges of slightly purulent urine, often caused by the passage of large sounds in the treatment of urethral strictures. In these cases there is pain after urination, similar to that which occurs in anal fissure after defecation. He asked whether these were not probably instances of pre-existing contracture of the vesical neck, whose mucous membrane has been actually fissured by the distending sound? Should this supposition be correct, would it not be proper to subject the more obstinate cases to the same sort of treatment as are cases of anal fissure? Anal fissure is frequently known to yield very speedily to divulsion of the sphincter, and it is more than probable that fissure of the vesical neck will, in many instances, yield to divulsion of the vesical neck. Divulsion of the urethro-vesical orifice can be made, among other means, by introducing *per urethram* a soft rubber tube capable of being distended, by water or air, to a given size and at a particular point. He has never resorted to internal incision for contracture of the vesical neck, as advised by the French. In some cases of chronic cystitis, from narrow intractable strictures, he has established free temporary drainage of the bladder, by first making external perineal urethrotomy, and then dilating the neck of the bladder by introducing the index finger. This has usually given excellent results. In only one case of cystitis from stricture has he made free incision of the neck of bladder, and this patient died.

In far advanced cases of cystitis, where the whole organ is implicated, and where there is concentric hypertrophy with diminished capacity, but where there is no reason to think that the kidneys are hopelessly involved, he advocated cystotomy, such as that recommended by Dr. Parker, of New York; but with the understanding that the cut shall be kept permanently open in order that the bladder may be constantly drained. He thought there could be no objection to this on the score of the inconvenience it might give the patient, since, in most cases that are likely to require the operation, no urine can be retained, and the patient may already be wearing a urinal to keep his garments dry—the bladder having ceased to act as a reservoir for the urine. The doctor stated that he had once succeeded in establishing such permanent drainage of the bladder. The operation was done three years ago, and the patient, who was before very feeble and emaciated, is now well and stout, and the only inconvenience he suffers is getting wet when his urinal is out of order.

He thought that the treatment of chronic cystitis should be in accord with the peculiarities of each case, and with the degree and cause of the disease, and that much judgment and experience are necessary to determine what should be done. While most cases are manageable by irrigations, etc., some are at first greatly damaged by this treatment, and require weeks of preparation by rest, diluents, balsamics, tonics, ice in the rectum, fomentations, hip-baths, rectal suppositories of belladonna and opium, etc. In the majority of instances the treatment should be constitutional as well as topical. The doctor enumerated the various causes of cystitis, and gave a brief outline of the treatment appropriate to each form.

In conclusion, he called attention to the importance of early treatment of the cystitis which so frequently follows injury or disease of the spinal cord, and said that for the past ten years it has been his practice, in injuries of the back, followed by paraplegia and paralysis of the bladder, not only to draw off the stagnant urine, but from the very first to frequently irrigate the bladder, twice and even thrice daily. This always gives ease and comfort to the patient, and often has much to do with the favorable termination of the case.—*Medical Record.*

